# 4-[(5-Chloro-3-methyl-1-phenyl-1*H*-pyrazol-4-yl)methyl­idene­amino]-1,5-dimethyl-2-phenyl-1*H*-pyrazol-3(2*H*)-one

**DOI:** 10.1107/S1600536810039048

**Published:** 2010-10-09

**Authors:** Hualing Zhu, Jinhua Zhu, Litong Ban, Pingping Zhang, Miao Zhang

**Affiliations:** a Department of Basic Science, Tianjin Agricultural College, Tianjin Jinjing Road No. 22, Tianjin 300384, People’s Republic of China; b Service Center of Meteorological Science and Technology of Shanxi Province, Taiyuan Xinjian Road No. 152, Taiyuan, Shanxi 030002, People’s Republic of China; c Department of Agricultural Science, Tianjin Agricultural College, Tianjin Jinjing Road No. 22, Tianjin 300384, People’s Republic of China; d Department of Food Science, Tianjin Agricultural College, Tianjin Jinjing Road No. 22, Tianjin 300384, People’s Republic of China

## Abstract

In the mol­ecule of the title compound, C_22_H_20_ClN_5_O, the atoms of the two pyrazole rings and the –C=N– group which joins them are essentially coplanar, with an r.m.s. deviation of 0.054 (2) Å. The phenyl rings form dihedral angles of 41.24 (5) and 55.53 (5)° with this plane. The crystal structure is stabilized by weak inter­molecular π–π inter­actions, with centroid-to-centroid distances of 3.6179 (13) Å between the imidazole rings.

## Related literature

For our previous work in this area, see: Zhu *et al.* (2005 ▶, 2010*a* ▶,*b* ▶,*c* ▶). For a related crystal structure, see: Shi *et al.* (2005 ▶).
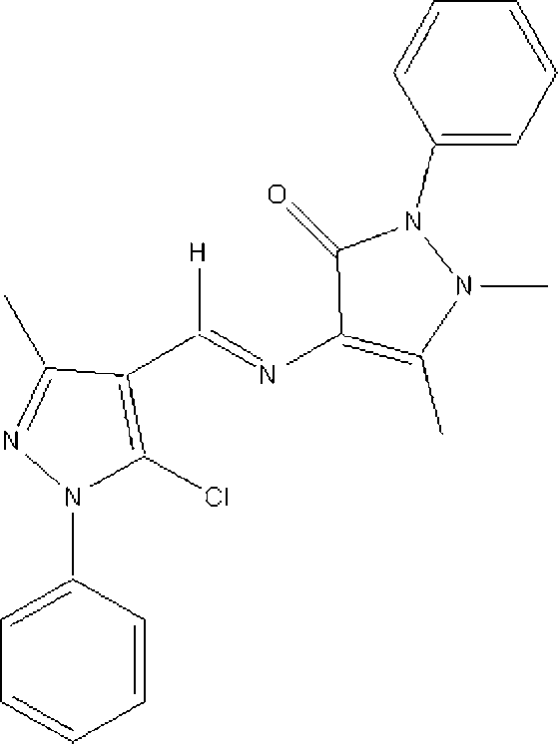


## Experimental

### 

#### Crystal data

C_22_H_20_ClN_5_O
*M* *_r_* = 405.88
Monoclinic, 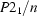

*a* = 8.3982 (17) Å
*b* = 9.5204 (19) Å
*c* = 24.401 (5) Å
β = 97.24 (3)°
*V* = 1935.4 (7) Å^3^
*Z* = 4
Mo *K*α radiation
μ = 0.22 mm^−1^
*T* = 113 K
0.20 × 0.18 × 0.10 mm

#### Data collection

Rigaku Saturn CCD diffractometer
Absorption correction: multi-scan (*CrystalClear*; Rigaku, 2008 ▶) *T*_min_ = 0.957, *T*_max_ = 0.978
15458 measured reflections
3397 independent reflections
2871 reflections with *I* > 2σ(*I*)
*R*_int_ = 0.046

#### Refinement

*R*[*F* ^2^ > 2σ(*F* ^2^)] = 0.044
*wR*(*F* ^2^) = 0.105
*S* = 1.08
3397 reflections
266 parameters
H-atom parameters constrained
Δρ_max_ = 0.41 e Å^−3^
Δρ_min_ = −0.25 e Å^−3^

### 

Data collection: *CrystalClear* (Rigaku, 2008 ▶); cell refinement: *CrystalClear*; data reduction: *CrystalClear*; program(s) used to solve structure: *SHELXS97* (Sheldrick, 2008 ▶); program(s) used to refine structure: *SHELXL97* (Sheldrick, 2008 ▶); molecular graphics: *ORTEP-3* (Farrugia, 1997 ▶) and Mercury (Macrae *et al.*, 2006 ▶); software used to prepare material for publication: *SHELXTL* (Sheldrick, 2008 ▶).

## Supplementary Material

Crystal structure: contains datablocks I, global. DOI: 10.1107/S1600536810039048/lh5137sup1.cif

Structure factors: contains datablocks I. DOI: 10.1107/S1600536810039048/lh5137Isup2.hkl

Additional supplementary materials:  crystallographic information; 3D view; checkCIF report

## Figures and Tables

**Fig. 1.**
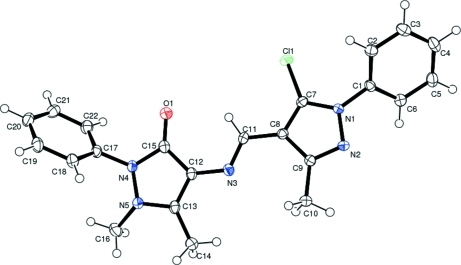
The molecular structure of the title compound with the atom numbering scheme. Displacement ellipsoids are drawn at the 30% probability level. H atoms are presented as a small spheres of arbitrary radius.

**Fig. 2.**
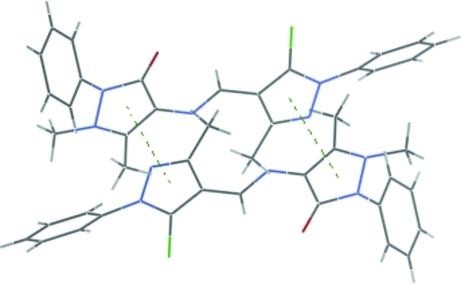
Part of the crystal structure showing intermolecular π–π interactions as dashed lines.

## supplementary crystallographic information

### Comment

As part of our ongoing studies (Zhu *et al.*, 2005, 2010*a*,*b*,*c*) of pyrazolone derivatives as potential ligands (Shi *et al.*, 2005) we report the crystal structure of the title compound (I).

The molecular structure of the title compound is shown in Fig. 1. Atoms C11 and N3 and the two pyrazole rings are essentially coplplanar with the largest deviation being 0.0543 (18) Å for atom C13. The crystal structure is stabilized by weak intermolecular π–π interactions with ring centroid to ring centroid distances of 3.6179 (13) Å between the imidazole rings (see Fig. 2).

### Experimental

The title compound was synthesized by refluxing a mixture of 4-formacyl-5-methyl-3-chloro-2-phenyl-2*H*-pyrazole (15*m* mol) and 4-antipyrine (15 mmol) in ethanol (100 ml) over a steam bath for about 4 h. The solution was then cooled to room temperature. After one day, pale-yellow blocks wre obtained and they were dried in air. Recrystallization of a solution of the title compound in ethanol afforded pale-yellow crystals suitable for X-ray analysis.

### Refinement

H atoms were placed in calculated positions with C—H = 0.95 and 0.98 Å and included in the refinement with U_iso_(H) = 1.2U_eq_(C) or 1.5U_eq_(C_methyl_).

### Crystal data

**Table:**

C_22_H_20_ClN_5_O	*F*(000) = 848
*M**_r_* = 405.88	*D*_x_ = 1.393 Mg m^−^^3^
Monoclinic, *P*2_1_/*n*	Mo *K*α radiation, λ = 0.71073 Å
Hall symbol: -P 2yn	Cell parameters from 4998 reflections
*a* = 8.3982 (17) Å	θ = 1.7–27.9°
*b* = 9.5204 (19) Å	µ = 0.22 mm^−^^1^
*c* = 24.401 (5) Å	*T* = 113 K
β = 97.24 (3)°	Block, pale yellow
*V* = 1935.4 (7) Å^3^	0.20 × 0.18 × 0.10 mm
*Z* = 4	

### Data collection

**Table:**

Rigaku Saturn CCD diffractometer	3397 independent reflections
Radiation source: rotating anode	2871 reflections with *I* > 2σ(*I*)
confocal	*R*_int_ = 0.046
Detector resolution: 7.31 pixels mm^-1^	θ_max_ = 25.0°, θ_min_ = 1.7°
ω and φ scans	*h* = −9→9
Absorption correction: multi-scan (*CrystalClear*; Rigaku, 2008)	*k* = −11→11
*T*_min_ = 0.957, *T*_max_ = 0.978	*l* = −29→29
15458 measured reflections	

### Refinement

**Table:**

Refinement on *F*^2^	Secondary atom site location: difference Fourier map
Least-squares matrix: full	Hydrogen site location: inferred from neighbouring sites
*R*[*F*^2^ > 2σ(*F*^2^)] = 0.044	H-atom parameters constrained
*wR*(*F*^2^) = 0.105	*w* = 1/[σ^2^(*F*_o_^2^) + (0.0533*P*)^2^ + 0.6515*P*] where *P* = (*F*_o_^2^ + 2*F*_c_^2^)/3
*S* = 1.08	(Δ/σ)_max_ = 0.001
3397 reflections	Δρ_max_ = 0.41 e Å^−^^3^
266 parameters	Δρ_min_ = −0.25 e Å^−^^3^
0 restraints	Extinction correction: *SHELXL97* (Sheldrick, 2008), Fc^*^=kFc[1+0.001xFc^2^λ^3^/sin(2θ)]^-1/4^
Primary atom site location: structure-invariant direct methods	Extinction coefficient: 0.0242 (16)

### Special details

**Table:**

Geometry. All e.s.d.'s (except the e.s.d. in the dihedral angle between two l.s. planes) are estimated using the full covariance matrix. The cell e.s.d.'s are taken into account individually in the estimation of e.s.d.'s in distances, angles and torsion angles; correlations between e.s.d.'s in cell parameters are only used when they are defined by crystal symmetry. An approximate (isotropic) treatment of cell e.s.d.'s is used for estimating e.s.d.'s involving l.s. planes.
Refinement. Refinement of *F*^2^ against ALL reflections. The weighted *R*-factor *wR* and goodness of fit *S* are based on *F*^2^, conventional *R*-factors *R* are based on *F*, with *F* set to zero for negative *F*^2^. The threshold expression of *F*^2^ > σ(*F*^2^) is used only for calculating *R*-factors(gt) *etc*. and is not relevant to the choice of reflections for refinement. *R*-factors based on *F*^2^ are statistically about twice as large as those based on *F*, and *R*- factors based on ALL data will be even larger.

### Fractional atomic coordinates and isotropic or equivalent isotropic displacement parameters (Å^2^)

**Table:**

	*x*	*y*	*z*	*U*_iso_*/*U*_eq_	
Cl1	0.56582 (6)	0.05661 (6)	0.08916 (2)	0.02960 (19)	
O1	0.75786 (16)	0.36105 (15)	−0.04914 (6)	0.0251 (4)	
N1	0.80398 (18)	−0.09683 (17)	0.14372 (6)	0.0175 (4)	
N2	0.96786 (17)	−0.11576 (16)	0.14899 (6)	0.0172 (4)	
N3	1.03166 (18)	0.19175 (16)	0.02437 (6)	0.0181 (4)	
N4	0.98109 (19)	0.45266 (17)	−0.08446 (6)	0.0196 (4)	
N5	1.14617 (18)	0.42156 (17)	−0.07913 (6)	0.0201 (4)	
C1	0.7142 (2)	−0.1746 (2)	0.17966 (8)	0.0181 (4)	
C2	0.5695 (2)	−0.2379 (2)	0.16005 (8)	0.0206 (4)	
H2	0.5247	−0.2262	0.1226	0.025*	
C3	0.4910 (2)	−0.3182 (2)	0.19566 (9)	0.0246 (5)	
H3	0.3912	−0.3610	0.1826	0.030*	
C4	0.5566 (2)	−0.3368 (2)	0.25005 (9)	0.0263 (5)	
H4	0.5026	−0.3927	0.2742	0.032*	
C5	0.7015 (2)	−0.2736 (2)	0.26924 (8)	0.0254 (5)	
H5	0.7469	−0.2867	0.3066	0.031*	
C6	0.7808 (2)	−0.1913 (2)	0.23432 (8)	0.0213 (5)	
H6	0.8794	−0.1468	0.2477	0.026*	
C7	0.7592 (2)	0.0000 (2)	0.10393 (8)	0.0186 (4)	
C8	0.8934 (2)	0.04644 (19)	0.08215 (8)	0.0164 (4)	
C9	1.0207 (2)	−0.0306 (2)	0.11224 (7)	0.0163 (4)	
C10	1.1952 (2)	−0.0254 (2)	0.10710 (8)	0.0209 (4)	
H10A	1.2521	−0.0946	0.1321	0.031*	
H10B	1.2366	0.0688	0.1168	0.031*	
H10C	1.2119	−0.0470	0.0690	0.031*	
C11	0.8978 (2)	0.14939 (19)	0.03858 (8)	0.0174 (4)	
H11	0.8001	0.1860	0.0203	0.021*	
C12	1.0353 (2)	0.2915 (2)	−0.01714 (8)	0.0178 (4)	
C13	1.1767 (2)	0.3331 (2)	−0.03503 (7)	0.0189 (4)	
C14	1.3431 (2)	0.2994 (2)	−0.01061 (8)	0.0265 (5)	
H14A	1.4097	0.2833	−0.0402	0.040*	
H14B	1.3425	0.2145	0.0121	0.040*	
H14C	1.3871	0.3779	0.0124	0.040*	
C15	0.9051 (2)	0.3662 (2)	−0.04929 (8)	0.0191 (4)	
C16	1.2558 (2)	0.5346 (2)	−0.09045 (9)	0.0277 (5)	
H16A	1.2703	0.5994	−0.0590	0.042*	
H16B	1.2106	0.5856	−0.1237	0.042*	
H16C	1.3598	0.4944	−0.0962	0.042*	
C17	0.9040 (2)	0.5093 (2)	−0.13518 (8)	0.0194 (4)	
C18	0.9457 (2)	0.4650 (2)	−0.18560 (8)	0.0231 (5)	
H18	1.0276	0.3968	−0.1870	0.028*	
C19	0.8665 (2)	0.5215 (2)	−0.23385 (8)	0.0257 (5)	
H19	0.8960	0.4932	−0.2685	0.031*	
C20	0.7448 (2)	0.6188 (2)	−0.23199 (8)	0.0250 (5)	
H20	0.6897	0.6560	−0.2652	0.030*	
C21	0.7038 (2)	0.6615 (2)	−0.18128 (9)	0.0256 (5)	
H21	0.6198	0.7278	−0.1799	0.031*	
C22	0.7844 (2)	0.6082 (2)	−0.13259 (8)	0.0235 (5)	
H22	0.7578	0.6393	−0.0979	0.028*	

### Atomic displacement parameters (Å^2^)

**Table:**

	*U*^11^	*U*^22^	*U*^33^	*U*^12^	*U*^13^	*U*^23^
Cl1	0.0146 (3)	0.0348 (3)	0.0391 (3)	0.0050 (2)	0.0026 (2)	0.0133 (2)
O1	0.0202 (8)	0.0284 (8)	0.0269 (8)	0.0014 (6)	0.0035 (6)	0.0068 (6)
N1	0.0137 (8)	0.0208 (9)	0.0185 (8)	−0.0001 (7)	0.0033 (6)	0.0039 (7)
N2	0.0132 (8)	0.0197 (9)	0.0187 (8)	0.0005 (7)	0.0021 (6)	0.0010 (7)
N3	0.0204 (9)	0.0170 (9)	0.0169 (8)	−0.0018 (7)	0.0024 (7)	0.0003 (6)
N4	0.0204 (8)	0.0215 (9)	0.0171 (8)	0.0023 (7)	0.0031 (7)	0.0051 (7)
N5	0.0189 (9)	0.0227 (9)	0.0185 (8)	−0.0020 (7)	0.0012 (7)	0.0040 (7)
C1	0.0181 (10)	0.0162 (10)	0.0213 (10)	0.0016 (8)	0.0073 (8)	0.0021 (8)
C2	0.0198 (10)	0.0218 (11)	0.0209 (10)	0.0011 (8)	0.0049 (8)	−0.0013 (8)
C3	0.0223 (11)	0.0198 (11)	0.0330 (12)	−0.0023 (8)	0.0087 (9)	−0.0020 (9)
C4	0.0305 (12)	0.0190 (11)	0.0324 (12)	0.0012 (9)	0.0155 (10)	0.0055 (9)
C5	0.0308 (12)	0.0253 (12)	0.0213 (10)	0.0037 (9)	0.0075 (9)	0.0055 (9)
C6	0.0211 (10)	0.0213 (11)	0.0215 (10)	−0.0002 (8)	0.0028 (8)	0.0018 (8)
C7	0.0152 (10)	0.0192 (11)	0.0208 (10)	0.0022 (8)	0.0006 (8)	0.0016 (8)
C8	0.0154 (9)	0.0168 (10)	0.0166 (9)	−0.0007 (8)	0.0010 (8)	−0.0004 (8)
C9	0.0170 (9)	0.0172 (10)	0.0148 (9)	−0.0010 (8)	0.0024 (8)	−0.0014 (8)
C10	0.0158 (10)	0.0248 (11)	0.0220 (10)	0.0005 (8)	0.0030 (8)	0.0026 (8)
C11	0.0192 (10)	0.0160 (10)	0.0166 (10)	0.0021 (8)	0.0001 (8)	0.0021 (8)
C12	0.0218 (10)	0.0162 (10)	0.0154 (9)	−0.0008 (8)	0.0021 (8)	0.0000 (8)
C13	0.0239 (10)	0.0199 (11)	0.0127 (9)	−0.0014 (8)	0.0012 (8)	−0.0008 (8)
C14	0.0213 (11)	0.0367 (13)	0.0217 (11)	−0.0007 (9)	0.0035 (9)	0.0048 (9)
C15	0.0231 (11)	0.0174 (11)	0.0169 (10)	−0.0015 (8)	0.0028 (8)	−0.0001 (8)
C16	0.0283 (12)	0.0251 (12)	0.0305 (12)	−0.0043 (9)	0.0072 (9)	0.0051 (9)
C17	0.0233 (10)	0.0162 (10)	0.0182 (10)	−0.0040 (8)	0.0001 (8)	0.0033 (8)
C18	0.0275 (11)	0.0207 (11)	0.0215 (10)	0.0009 (9)	0.0054 (9)	0.0028 (8)
C19	0.0334 (12)	0.0276 (12)	0.0160 (10)	−0.0055 (10)	0.0030 (9)	0.0006 (9)
C20	0.0279 (11)	0.0231 (12)	0.0219 (10)	−0.0055 (9)	−0.0048 (9)	0.0046 (9)
C21	0.0239 (11)	0.0221 (11)	0.0293 (12)	0.0015 (9)	−0.0025 (9)	0.0016 (9)
C22	0.0277 (11)	0.0228 (11)	0.0198 (10)	−0.0011 (9)	0.0019 (9)	−0.0013 (8)

### Geometric parameters (Å, °)

**Table:**

Cl1—C7	1.7064 (19)	C8—C11	1.450 (3)
O1—C15	1.238 (2)	C9—C10	1.487 (3)
N1—C7	1.357 (2)	C10—H10A	0.9800
N1—N2	1.378 (2)	C10—H10B	0.9800
N1—C1	1.433 (2)	C10—H10C	0.9800
N2—C9	1.326 (2)	C11—H11	0.9500
N3—C11	1.282 (2)	C12—C13	1.374 (3)
N3—C12	1.392 (2)	C12—C15	1.449 (3)
N4—C15	1.400 (2)	C13—C14	1.484 (3)
N4—N5	1.408 (2)	C14—H14A	0.9800
N4—C17	1.428 (2)	C14—H14B	0.9800
N5—C13	1.365 (2)	C14—H14C	0.9800
N5—C16	1.465 (3)	C16—H16A	0.9800
C1—C2	1.386 (3)	C16—H16B	0.9800
C1—C6	1.389 (3)	C16—H16C	0.9800
C2—C3	1.385 (3)	C17—C22	1.384 (3)
C2—H2	0.9500	C17—C18	1.387 (3)
C3—C4	1.383 (3)	C18—C19	1.385 (3)
C3—H3	0.9500	C18—H18	0.9500
C4—C5	1.385 (3)	C19—C20	1.385 (3)
C4—H4	0.9500	C19—H19	0.9500
C5—C6	1.388 (3)	C20—C21	1.387 (3)
C5—H5	0.9500	C20—H20	0.9500
C6—H6	0.9500	C21—C22	1.387 (3)
C7—C8	1.378 (3)	C21—H21	0.9500
C8—C9	1.422 (3)	C22—H22	0.9500
			
C7—N1—N2	109.82 (14)	H10B—C10—H10C	109.5
C7—N1—C1	132.03 (16)	N3—C11—C8	121.01 (17)
N2—N1—C1	118.13 (15)	N3—C11—H11	119.5
C9—N2—N1	105.80 (15)	C8—C11—H11	119.5
C11—N3—C12	120.78 (16)	C13—C12—N3	121.80 (17)
C15—N4—N5	109.84 (15)	C13—C12—C15	108.06 (17)
C15—N4—C17	124.14 (16)	N3—C12—C15	130.13 (17)
N5—N4—C17	119.57 (15)	N5—C13—C12	110.13 (17)
C13—N5—N4	106.72 (14)	N5—C13—C14	121.60 (17)
C13—N5—C16	122.81 (16)	C12—C13—C14	128.17 (18)
N4—N5—C16	117.58 (15)	C13—C14—H14A	109.5
C2—C1—C6	120.76 (18)	C13—C14—H14B	109.5
C2—C1—N1	121.27 (17)	H14A—C14—H14B	109.5
C6—C1—N1	117.88 (17)	C13—C14—H14C	109.5
C3—C2—C1	119.28 (19)	H14A—C14—H14C	109.5
C3—C2—H2	120.4	H14B—C14—H14C	109.5
C1—C2—H2	120.4	O1—C15—N4	123.90 (17)
C4—C3—C2	120.61 (19)	O1—C15—C12	131.67 (18)
C4—C3—H3	119.7	N4—C15—C12	104.43 (16)
C2—C3—H3	119.7	N5—C16—H16A	109.5
C3—C4—C5	119.72 (19)	N5—C16—H16B	109.5
C3—C4—H4	120.1	H16A—C16—H16B	109.5
C5—C4—H4	120.1	N5—C16—H16C	109.5
C4—C5—C6	120.45 (19)	H16A—C16—H16C	109.5
C4—C5—H5	119.8	H16B—C16—H16C	109.5
C6—C5—H5	119.8	C22—C17—C18	120.90 (18)
C5—C6—C1	119.18 (18)	C22—C17—N4	118.02 (17)
C5—C6—H6	120.4	C18—C17—N4	121.07 (18)
C1—C6—H6	120.4	C19—C18—C17	119.21 (19)
N1—C7—C8	109.14 (16)	C19—C18—H18	120.4
N1—C7—Cl1	122.43 (14)	C17—C18—H18	120.4
C8—C7—Cl1	128.31 (15)	C20—C19—C18	120.59 (19)
C7—C8—C9	103.49 (16)	C20—C19—H19	119.7
C7—C8—C11	126.70 (17)	C18—C19—H19	119.7
C9—C8—C11	129.81 (17)	C19—C20—C21	119.54 (19)
N2—C9—C8	111.75 (16)	C19—C20—H20	120.2
N2—C9—C10	119.64 (17)	C21—C20—H20	120.2
C8—C9—C10	128.61 (17)	C20—C21—C22	120.50 (19)
C9—C10—H10A	109.5	C20—C21—H21	119.8
C9—C10—H10B	109.5	C22—C21—H21	119.8
H10A—C10—H10B	109.5	C17—C22—C21	119.24 (19)
C9—C10—H10C	109.5	C17—C22—H22	120.4
H10A—C10—H10C	109.5	C21—C22—H22	120.4
			
C7—N1—N2—C9	−0.2 (2)	C7—C8—C11—N3	175.01 (19)
C1—N1—N2—C9	−178.74 (16)	C9—C8—C11—N3	−5.4 (3)
C15—N4—N5—C13	−9.4 (2)	C11—N3—C12—C13	−176.57 (18)
C17—N4—N5—C13	−162.32 (16)	C11—N3—C12—C15	2.0 (3)
C15—N4—N5—C16	−152.04 (17)	N4—N5—C13—C12	8.4 (2)
C17—N4—N5—C16	55.0 (2)	C16—N5—C13—C12	148.65 (18)
C7—N1—C1—C2	44.7 (3)	N4—N5—C13—C14	−168.34 (17)
N2—N1—C1—C2	−137.15 (18)	C16—N5—C13—C14	−28.1 (3)
C7—N1—C1—C6	−138.7 (2)	N3—C12—C13—N5	174.47 (16)
N2—N1—C1—C6	39.5 (2)	C15—C12—C13—N5	−4.4 (2)
C6—C1—C2—C3	0.1 (3)	N3—C12—C13—C14	−9.1 (3)
N1—C1—C2—C3	176.61 (17)	C15—C12—C13—C14	172.05 (19)
C1—C2—C3—C4	−0.7 (3)	N5—N4—C15—O1	−172.85 (17)
C2—C3—C4—C5	0.5 (3)	C17—N4—C15—O1	−21.4 (3)
C3—C4—C5—C6	0.3 (3)	N5—N4—C15—C12	6.6 (2)
C4—C5—C6—C1	−0.9 (3)	C17—N4—C15—C12	158.02 (17)
C2—C1—C6—C5	0.7 (3)	C13—C12—C15—O1	177.9 (2)
N1—C1—C6—C5	−175.93 (17)	N3—C12—C15—O1	−0.8 (4)
N2—N1—C7—C8	0.1 (2)	C13—C12—C15—N4	−1.4 (2)
C1—N1—C7—C8	178.39 (18)	N3—C12—C15—N4	179.85 (18)
N2—N1—C7—Cl1	−176.18 (13)	C15—N4—C17—C22	67.4 (3)
C1—N1—C7—Cl1	2.1 (3)	N5—N4—C17—C22	−143.73 (18)
N1—C7—C8—C9	0.0 (2)	C15—N4—C17—C18	−111.6 (2)
Cl1—C7—C8—C9	176.01 (15)	N5—N4—C17—C18	37.3 (3)
N1—C7—C8—C11	179.71 (17)	C22—C17—C18—C19	0.3 (3)
Cl1—C7—C8—C11	−4.3 (3)	N4—C17—C18—C19	179.23 (18)
N1—N2—C9—C8	0.2 (2)	C17—C18—C19—C20	−1.4 (3)
N1—N2—C9—C10	−179.94 (16)	C18—C19—C20—C21	1.0 (3)
C7—C8—C9—N2	−0.1 (2)	C19—C20—C21—C22	0.4 (3)
C11—C8—C9—N2	−179.82 (18)	C18—C17—C22—C21	1.1 (3)
C7—C8—C9—C10	−179.97 (19)	N4—C17—C22—C21	−177.85 (17)
C11—C8—C9—C10	0.3 (3)	C20—C21—C22—C17	−1.5 (3)
C12—N3—C11—C8	−179.42 (16)		
